# Dynamic Layered Dual-Cluster Heads Routing Algorithm Based on Krill Herd Optimization in UWSNs

**DOI:** 10.3390/s16091379

**Published:** 2016-08-29

**Authors:** Peng Jiang, Yang Feng, Feng Wu, Shanen Yu, Huan Xu

**Affiliations:** College of Automation, Hangzhou Dianzi University, Hangzhou 310018, China; 141060021@hdu.edu.cn (Y.F.); fengwu@hdu.edu.cn (F.W.); shanen_yu@hdu.edu.cn (S.Y.); xuhuan@hdu.edu.cn (H.X.)

**Keywords:** UWSNs, routing algorithm, Krill Herd optimization, dynamic layered

## Abstract

Aimed at the limited energy of nodes in underwater wireless sensor networks (UWSNs) and the heavy load of cluster heads in clustering routing algorithms, this paper proposes a dynamic layered dual-cluster routing algorithm based on Krill Herd optimization in UWSNs. Cluster size is first decided by the distance between the cluster head nodes and sink node, and a dynamic layered mechanism is established to avoid the repeated selection of the same cluster head nodes. Using Krill Herd optimization algorithm selects the optimal and second optimal cluster heads, and its Lagrange model directs nodes to a high likelihood area. It ultimately realizes the functions of data collection and data transition. The simulation results show that the proposed algorithm can effectively decrease cluster energy consumption, balance the network energy consumption, and prolong the network lifetime.

## 1. Introduction

Underwater wireless sensor networks (UWSNs) are network monitoring systems which consist of sensor nodes. They can achieve acoustic communication and computation in underwater environment. UWSNs have been used in the fields of water environment monitoring, strategic surveillance, and underwater exploration. Thus, they gain increasing attention from governments and research institutions in various countries. There is no doubt that the UWSNs become a popular research topic today [1]. Sensor nodes use their own battery to provide limited energy. When they stop working and run out of energy, network topology is affected. As such, energy saving becomes a key issue in the research of UWSNs.

The design of routing algorithm whose function is to balance energy consumption and prolong the lifetime of networks in UWSNs also becomes a necessity.

Traditional routing algorithms can be divided into plane routing algorithms and clustering routing algorithms [2,3,4]. The latter is widely preferred than the former because of its satisfactory performance in terms of energy saving [5]. As early as 2000, Heinzelman et al. proposed low-energy adaptive clustering hierarchy (LEACH) algorithm. The cluster heads of LEACH transport data to the base station in a single hop communication manner. This leads to a large amount of information to nodes from the base station, so nodes face “premature death” due to running out of energy. Shang et al. proposed the distributed clustering routing algorithm. The algorithm makes the cluster heads generate further rationalization, but a large amount of information exchange leads to the loss of extra energy.

As a computing technology that can quickly and efficiently solve complex problems, swarm intelligence has been applied to UWSNs clustering routing algorithm [6]. Swarm intelligence has various techniques, such as the cat swarm optimization algorithm (CSO), the fish swarm algorithm, the ant colony algorithm (ACO), and the particle swarm optimization (PSO) algorithm, among others [7,8,9,10,11]. In general, swarm intelligence is easy to understand, has low complexity and strong commonality. Zhang et al. [7] proposed a clustering routing algorithm based on type-2 fuzzy logic and ACO. In particular, the type-2 fuzzy logic was used to solve the network uncertainty and balance the energy load. ACO was adopted to select the candidate cluster head to reduce the energy consumption. The algorithm presented by these researchers can prolong the network survival time. ACO is constructed solution path by a number of ants together. All of the ants improve the quality of the solution through the legacy and exchange of information in the solution path, and then it achieves the goals of optimization. As a general stochastic optimization method, ant colony algorithm has already been used successfully in a series of combinatorial optimization problems. It also achieved perfect results. However, because the algorithm is a typical probability algorithm, the parameters set in the algorithm is usually determined by experimental methods. This leads to the optimization of the performance that is closely related to people’s experience. Thus, it is difficult to optimize the algorithm performance.

Kong et al. [8] developed an energy-aware routing algorithm based on cat swarm optimization (CSO). The design of the proposed algorithm was based on a ladder diffusion algorithm to avoid the generation of circle routes but to provide backup routes. In addition, CSO was integrated to effectively provide improved efficiency and reduce the execution time for finding the routing path. The cat swarm algorithm uses the combination rate of the search mode and tracking mode to solve complex optimization problems. The convergence speed of the cat swarm algorithm remains to be further improved.

Xie et al. [9] introduced a new dual-cluster heads clustering routing algorithm based on PSO (DC-PSO). This algorithm can share the energy consumption of cluster heads by setting the cluster routing algorithm. However, a great amount of data transmission is produced when the clusters are near the base station. This leads to the premature death of the cluster head nodes. Therefore, the algorithm cannot fundamentally solve the “hot spots”. It is obvious that different swarm intelligence algorithms have their unique advantages [12]. Compared with traditional intelligent algorithms, the later developed algorithms perform better. In general, selecting and improving a new swarm intelligence algorithm has practical research significance. Our goal is to effectively solve the problem of clustering routing quickly.

To improve the utilization of network energy, this paper proposes dynamic layered dual-cluster heads routing algorithm based on Krill Herd (KH) optimization in UWSNs. When the distance between sink nodes is minimal, a huge amount of energy is consumed. In this case, the underwater network sets the non-uniform clusters according to the size of the clusters and the distance between nodes and the sink node. It can reduce the premature death of the upper nodes. The dynamic hierarchical mechanism is introduced. It can reduce the cluster head nodes that are repeatedly selected by the same node. Meanwhile, the network energy consumption is balanced. Finally, the core of the KH optimization is used to choose the master cluster head nodes and vice cluster head nodes. It solves the problem that the cluster head nodes are under heavy load, and effectively prolongs the survival time of cluster nodes.

## 2. Models and Definitions

### 2.1. Network Model

Sensor nodes are assumed to be randomly distributed in a 3D underwater monitoring space, and the sink node is placed in the water. Owing to the existence of various underwater network models, this study also considers other assumptions to achieve network function. These assumptions are as follows:

1. Each node has a unique ID number, and data are received successfully as soon as the information is passed to the sink node.
2. Each node except for the sink node has abilities of communication and mobility.
3. The nodes collect data cyclically. The sink receives information all the time.

### 2.2. Underwater Acoustic Energy Consumption Model

UWSNs vary from terrestrial wireless sensor network because they adopt a unique manner of underwater acoustic communication. Therefore, considerable research has looked into the energy consumption model to adapt the underwater environments. To develop an underwater acoustic energy consumption model, the underwater acoustic signal attenuation model A(d) is expressed as follows:

(1) $$A\left(d\right)=d^{q}\cdot a^{d}$$

where *q* is the diffusion factor, and it usually sets 1.5.

(2) $$10\text{log}\;a\left(f\right)=\frac{0.11\times f^{2}}{1+f^{2}}+\frac{44\times f^{2}}{4100+f^{2}}+2.75\times 10^{-4}\times f^{2}+0.003$$

where Equation (2) represents a=10a(f)/10. In this case, a is determined by the absorption coefficient a(f) in dB/m and *f* is the carrier frequency in kHz.

The information transmission distance between a node and the other node is far from d meters. The node energy consumption is depicted as follows:

(3) $$E=E_{send}+E_{rec}+E_{int}$$

(4) $$E_{send}=lP_{0}A\left(d\right)$$

(5) $$E_{rec}=lP_{r}$$

(6) $$E_{int}=lE_{da}$$

where $E_{send}$, $E_{rec}$ and $E_{int}$ are the energy consumption of the transmitting data, received data, and data fusion respectively. $P_{r}$ normally sets as a constant, it is the energy consumption of each unit. l is the data packet, and $P_{0}$ is the minimum power required for the underwater node to receive unit information, and $E_{da}$ is the energy consumption which compresses every package.

## 3. Problem and Algorithm Description

### 3.1. Problem Description

The clustering routing algorithm in UWSNs generally forwards data from the cluster nodes to each cluster head node. The data are sent to the sink node through the manner of single or multiple hops, thereby leading to a higher energy consumption of the cluster head node than other nodes in the cluster. This discrepancy in consumption amount causes the unbalanced energy consumption of the network. It also leads to the premature death of cluster head nodes. To solve this problem, Xie et al. [9] proposed DC-PSO. Although dual-cluster head and cluster multiple-hop routing methods can effectively reduce the energy consumption of cluster heads, they fail to fundamentally solve the problem of “hot zones”.

Considering the problems cited in the preceding paragraph, this study sets clusters according to the distance between the cluster and base station. If the distance is large, the numbers of cluster are smaller. The purpose is to share the cluster head nodes’ energy. In addition, the dynamic hierarchical mechanism is to avoid that the same node that is repeatedly selected as the cluster head. Krill swarm optimization is then applied to the master cluster heads and vice-cluster heads in the selection process. In sum, this paper proposes a dynamic layered dual-cluster heads routing algorithm based on KH optimization.

### 3.2. Krill Swarm Optimization Algorithm

Gandomi and Alavi proposed a KH optimization algorithm in the “Communications in Nonlinear Science and Numerical Simulation”; that is, a new bio-inspired optimization algorithm based on the simulation of the herding behavior of krill individuals [13]. In this algorithm, the krill moves to its location oriented with a high likelihood region using Lagrangian model, which mainly considers the distance from the food and from the highest density of the krill swarm. The movement of krill individuals includes three main actions: the movement induced by other krill individuals ($N_{i}$), foraging activity ($F_{i}$), and random diffusion ($D_{i}$). These moving actions interact iteratively and update their location until the global optimal solution is obtained.

1. The following Lagrangian model is generalized to an n-dimensional decision space:
  (7) $$\frac{dx_{i}}{dt}=N_{i}+D_{i}+F_{i}$$
2. Movement Induced by Other Krill Individuals

Krill individuals try to maintain a high density and move due to their mutual effects. For an individual krill, this movement can be defined as follows:

(8) $$N_{i}^{new}=N^{max}d_{i}+w_{n}N_{i}^{old}$$

(9) $$d_{i}=d_{i}^{local}+d_{i}^{target}$$

where $N^{max}$ is the maximum induced speed which set to 0.01 (ms^−1^), $d_{i}$ is estimated from the local swarm density (local effect), $w_{n}$ is the inertia weight of the motion which is distributed in the range [0, 1], $N_{i}^{old}$ is the last induced motion, $d_{i}^{local}$ is the local effect provided by the neighbors, and $d_{i}^{target}$ is the target direction effect provided by the best krill individual.

The effect of the neighbors can be assumed as attractive and repulsive tendency between the individuals for a local search. In this study, the effect of the neighbors on individual krill movement individual is determined with the following equations:

(10) $$d_{i}^{local}=\overset{NN}{\underset{j=1}{\sum }}K_{i,j}X_{i,j}$$

(11) $$X_{i,j}=\frac{X_{j}-X_{i}}{\left\|X_{j}-X_{i}\right\|+\varepsilon }$$

(12) $$K_{i,j}=\frac{K_{j}-K_{i}}{K^{worst}-K^{best}}$$

To avoid singularities, a small positive number *ε* is added to the denominator. *NN* is the number of the neighbors, $K_{i}$ represents the fitness or the objective function value of the *i*-th krill individual, $K_{j}$ is the fitness of *j*-th (*j* = 1, 2, …, *NN*), $K^{best}$ and $K^{worst}$ are the best fitness and the worst fitness values of the krill individuals respectively, and *X* represents the related positions.

According to the actual behavior of krill, the neighbors can be found by other krill individual once the sensing distance is determined. The expression for this condition is as follows:

(13) $$d_{s,i}=\frac{1}{5N}\overset{N}{\underset{j=1}{\sum }}\left\|X_{j}-X_{i}\right\|$$

where *d_s,i_* is the sensing distance for the *i*-th krill individual, and *N* is the number of krill individuals. If the distance of two krill individuals is less than the defined sensing distance, then they are neighbors.

Considering the effect of the individual krill with the best fitness, the following equation can be obtained:

(14) $$d_{i}^{target}=C^{best}K_{i,best}X_{i,best}$$

(15) $$C^{best}=2\left(rand+\frac{I}{I_{max}}\right)$$

where $C^{best}$ is the effective coefficient of the krill individual with the best fitness, rand is the random values between 0 and 1 which is for enhancing exploration, I is the actual iteration number, and $I_{max}$ is the maximum number of iterations.

3. Foraging Motion

The foraging motion of the krill individuals is formulated in terms of two main effective parameters. The first one is the food location. The second is the previous experience about the food location. These parameters are depicted below.

(16) $$F_{i}=v_{f}\;\beta_{i}+w_{f}F_{i}^{old}$$

where $F_{i}^{old}$ is the old foraging motion.

(17) $$\beta_{i}=\beta_{i}^{food}+\beta_{i}^{best}$$

where *v_f_* is the foraging speed which sets to 0.02 (ms^−1^), $w_{f}$ is the inertia weight of the foraging motion which distributes in the range [0, 1], $\beta_{i}^{food}$ is the food attractive, and $\beta_{i}^{best}$ is the effect of the best fitness.

Food effect is defined in terms of its location. The center of food should first be identified. It is followed by the formulation of food attraction. Food effect cannot be determined but can be estimated. In this study, the virtual center of food concentration is estimated according to the fitness distribution of the krill individuals inspired from the “center of mass”. The formula for this variable is as follows:

(18) $$X^{food}=\frac{\sum_{i=1}^{N}\;\frac{1}{K_{i}}\;X_{i}}{\sum_{i=1}^{N}\;\frac{1}{K_{i}}}$$

Therefore, the food attraction for the *i*-th krill individual can be determined using the following equation:

(19) $$\beta_{i}^{food}=C^{food}K_{i,food}X_{i,food}$$

(20) $$C^{food}=2\times \left(1-\frac{I}{I_{max}}\right)$$

where $C^{food}$ is the food coefficient. The effect of food on KH decreases with time.

The foraging motion of individual krill promotes global optimization, and krill individuals normally herd around the global optima after a number of iterations. Therefore, this proceeding can be considered an efficient global optimization strategy that can improve the globality of the KH algorithm.

The effect of the best fitness of the *i*-th krill individual is identified using the following equation:

(21) $$\beta_{i}^{best}=K_{i,ibest}X_{i,ibest}$$

where $K_{i,ibest}$ is the best previously visited position of the *i*-th krill individual.

4. Random Diffusion

Random motion can be expressed in terms of a maximum diffusion speed and a random directional vector. This variable can be formulated as follows:

(22) $$D_{i}=D^{max}\left(1-\frac{I}{I_{max}}\right)\delta$$

where $D^{max}$ is the maximum diffusion speed, and *δ* is the random directional vector whose arrays are random values varying between −1 and 1.

5. Status Update

The status update can be formulated as follows:

(23) $$X_{i}^{\left(n+1\right)}=X_{i}^{\left(n\right)}+\left(N_{i}^{new}+F_{i}^{new}+D_{i}^{new}\right)\cdot ∆t$$

where ∆t is the time interval and should be carefully set according to actual situations, and $N_{i}^{new}$ is the newest induced motion speed, $F_{i}^{new}$ is the newest foraging motion speed, $D_{i}^{new}$ is the newest diffusion speed.

The parameters of krill swarm optimization algorithm are based on the actual simulation of krill motion and experimental validation. Among these parameters, only the one-time interval parameters need to be adjusted automatically. This case is one of the characteristics of the intelligent algorithm that makes it better than other groups. In this study, we attempt to improve the krill swarm optimization algorithm to solve the routing problem of UWSNs.

### 3.3. DC-KH Algorithm Description

#### 3.3.1. Dynamic Hierarchical and Non-Uniform Clustering Stage

At first, UWSNs are initialized. Then, a random number (ζ) varying between 0 and 1 is assigned to each node. The relationship between the size of ζ and the threshold (τ) is judged. If ζ is less than τ, then the node candidate is the master cluster head node. Otherwise, it becomes the node in the cluster until the end of cluster head election. To solve the problem of “hot spots”, the non-uniform clustering of UWSNs can be realized in the latter stage by setting the competition radius [14]. It is formulated as follows:

(24) $$\tau =\frac{p}{\left[1-\text{pr}\cdot \text{mod}\left(\frac{1}{p}\right)\right]}\cdot \frac{E\left(n_{i}\right)}{E_{n\_max}},\;n\in G$$

(25) $$R_{c}=\left[1-\frac{c\left(d_{max}-d_{i\to Bs}\right)}{d_{max}-d_{min}}\right]\cdot R_{c}^{max}$$

where in Equation (24), *p* is the probability of being selected as the cluster head, *r* is the number of cycles, $E_{n\_max}$ is the initial energy, $E\left(n_{i}\right)$ is the current energy, and *G* is the current cluster head node set. In Equation (25), $d_{max}$ and $d_{min}$ are, respectively, the farthest and nearest distance between the node and base station, $d_{i\to Bs}$ is the distance between candidate cluster head and base station, $R_{c}^{max}$ is the maximum competitive radius, and *c* is a random value ranging from 0 to 1.

To avoid the nodes being repeatedly selected as cluster heads, all nodes are dynamically stratified. The nodes are divided into H/(R_t_ + 1) layers, and each layer is numbered from 1. H is the depth of the water, and d is a fixed value. After the R_t_/d round again, the network is restored to the initial state of stratification. Given that the *0*-th layer node is in the range of sink nodes, the cluster cannot be formed. In this case, the first round of the first floor is the ratio of water depth (∆d) and interval. At the end of each round, the network layer will adjust the distance (d) downwards. Equation (26) represents the nodes in the first round of the hierarchy.

(26) $$L_{ni}=\left[\frac{h_{i}+∆d\cdot mod\left(n-1,∆d/d\right)}{∆d}\right]$$

**Figure 1:**
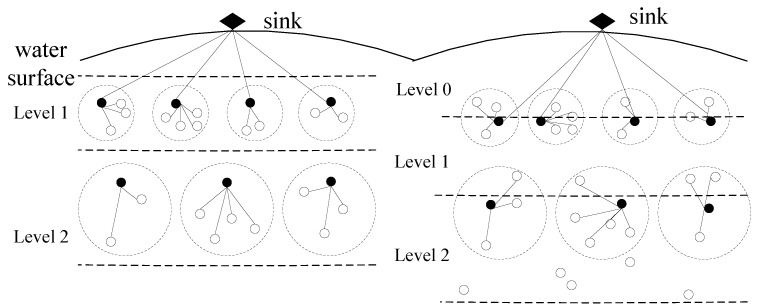
Sketch map of node dynamic hierarchical and non-uniform node clustering.

Figure 1 is a sketch map of the node dynamic hierarchical and non-uniform clustering. The figure shows that suitable cluster heads are selected by dynamic hierarchical and non-uniform clustering. Thus, it solves the problem of premature death of cluster head nodes and the network “hot spots”. In addition, the network energy consumption can be balanced, and the survival time of UWSNs can be prolonged.

#### 3.3.2. KH Main Cluster Head and Vice-Cluster Head Selection Phase

When the dynamic layer and non-uniform clustering stage is completed, the master cluster heads and vice-cluster heads are selected with the improved KH algorithm. First of all, a threshold is set. Once the radius of the cluster is less than this threshold, the candidate cluster head becomes the cluster head. On the contrary, the two cluster heads are selected in the first phase according to the KH algorithm.

1. Fitness Function

The performance of cluster head in the cluster routing algorithm depends on the choice of the fitness function. To prolong the survival time of the master cluster head, the condition that the energy loss of the main cluster head is significantly more than that of the ordinary node should be considered. The selection of the location of the master cluster head should be considered as well to collect the information of other nodes in cluster. The distance between the two nodes is hoped to reach the minimum. To select the optimal cluster head, the formula of the adaptive function shown below is adopted:

(27) $$f=\varepsilon \times f_{1}+\left(1-\varepsilon \right)f_{2}$$

(28) $$f_{1}=E\left(H\right)/\overset{m}{\underset{i=1}{\sum }}E\left(n_{i}\right)$$

(29) $$f_{2}=\left(m-1\right)/\overset{m}{\underset{i=1}{\sum }}d_{i\to H}$$

where $f_{1}$ is the ratio of cluster head node to total energy, the reciprocal of $f_{2}$ is the average distance between the two, *m* is the total number of nodes in a cluster, $E\left(n_{i}\right)$ is the energy of the $n_{i}$ node in the cluster, and $d_{i\to H}$ is the distance from the main cluster head to node N.

According to the main cluster head fitness function, the values of *ε* for controlling the proportions of *f_1_* and *f_2_* should be adjusted so that the maximum value of *f* is the optimal cluster head. The sub-cluster head should then be selected according to the principle of distance from the base station mainly to achieve two purposes: the distance from the base station should be near and must have high energy. The fitness function can be denoted as follows:

(30) $$g=\lambda g_{1}+\left(1-\lambda \right)g_{2}$$

(31) $$g_{1}=E\left(H\right)/\overset{m}{\underset{i=1}{\sum }}E\left(n_{i}\right)$$

(32) $$g_{2}=d_{H\to BS}/\overset{m}{\underset{i=1}{\sum }}d_{i\to BS}$$

where *g_1_* is the ratio of cluster head node to total energy, and $d_{H\to BS}$ is the distance between the vice-cluster head and base station. $\sum_{i=1}^{m}d_{i\to BS}$ is the sum of the distances between all nodes in cluster and base station; and $g_{2}$ is the ratio between the two nodes. The method is consistent with the master cluster head selection method. Once *g* is the largest among all variables, the optimal pair is selected as the optimal sub-cluster head.

2. Dual-Cluster Head Selection

To select the master cluster heads and vice cluster heads, Krill Herd optimization algorithm is improved. Given that the node deployment environment is three-dimensional, underwater, its location is determined using three components in x, y, and z on the coordinate axis. Therefore, Equation (23) is represented by the group of Equations from (33) to (35):

(33) $$X_{ix}^{\left(n+1\right)}=X_{ix}^{\left(n\right)}+\left[F_{i}^{new}+N_{i}^{new}+D_{i}^{new}\right]\cdot ∆t$$

(34) $$X_{iy}^{\left(n+1\right)}=X_{iy}^{\left(n\right)}+\left[F_{i}^{new}+N_{i}^{new}+D_{i}^{new}\right]\cdot ∆t$$

(35) $$X_{iz}^{\left(n+1\right)}=X_{iz}^{\left(n\right)}+\left[F_{i}^{new}+N_{i}^{new}+D_{i}^{new}\right]\cdot ∆t$$

Considering that the nodes in the water are distributed discretely, the calculated value of the above formula cannot be mapped directly onto the location of the actual node. Thus, the following adjustments are made to the cluster node location:

(36) $$∆p_{i}=\sqrt{{\left(∆p_{ix}\right)}^{2}+{\left(∆p_{iy}\right)}^{2}+{\left(∆p_{iy}\right)}^{2}}$$

(37) $$∆p_{k}=\text{min}\left(∆p_{1},∆p_{2},\ldots ,∆p_{n-1},∆p_{n}\right)$$

(38) $$x_{id}\left(n\right)\approx p_{k}$$

where $∆p_{ix}$, $∆p_{iy}$, and $∆p_{iz}$are the absolute values of the components of *x*, *y*, and *z* in the cluster, respectively, $∆p_{k}$ is the position that mostly fits the actual situation, and $x_{i}\left(n\right)$ is the adjusted node position.

Having reached this point, we can confirm that a dynamic layered dual-cluster routing algorithm based on Krill Herd optimization in UWSNs is accomplished. These steps are enumerated as follows:

Step 1: Initialization of krill. Each individual krill random location in 3D space should be determined, followed by the adjustment of the position and its mapping onto the node distribution in water. This step is accomplished with Equations (36)–(38).
Step 2: Calculation of the fitness value. The current position of krill is calculated within the clusters krill individual extremum and the maximum adaptation values. The krill location is the krill swarm global extremum. Equations (27)–(29) are used for this step.
Step 3: Update and adjust the position. Equations (36)–(38) are employed to adjust the existing position of krill.
Step 4: The updated adaptation value is calculated, and the global and local extremums are updated with Equations (27)–(29).
Step 5: Steps 3 and 4 should be repeated prior to reaching the maximum number of iterations.
Step 6: The global extremum is selected as the master cluster head.
Step 7: Using the vice cluster head, the value function Equation (30) is fitted to Equation (32). The preceding step is repeated to remove the vice-cluster head.

#### 3.3.3. Single and Multi-Hop Transmission

The vice-cluster head is mainly responsible for transmitting the information of the main cluster head to the sink node through the manner of single hop or multiple hops. In the initial stage, the vice-cluster head broadcasts its own ID number, the remaining energy, and so on. If A receives the message of B, and it selects A as the vice cluster head, then B sends L bit data to the sink node. Equation (3) is used to calculate the energy consumption model. To ensure the communication overhead and energy of the next hop node, the weight of the sub cluster head is calculated with Equation (39).

(39) $$W\left(i\right)=\partial \cdot \left[\frac{E\left(H_{j}\right)}{E\left(H_{i}\right)}\right]+\left(1-\partial \right)\cdot \left[d_{i\to BS}^{4}/\left(d_{i\to j}^{2}+d_{j\to BS}^{2}\right)\right]$$

where ∂ is determined according to actual situation. If *W*(*i*) is greater than 1, then the vice-cluster head is selected as the maximum next hop node. The vice-cluster head then directly sends the data to the sink node.

## 4. Simulation

In the experiment, the UWSN’s node deployment process is simulated using MATLAB (Natick, MA, USA) based on the background of Xixi Wetland water environment monitoring. During the simulation, the target water area (length × width × depth) is set to (150 × 150 × 150) m^3^. At the initial time, 200 nodes are randomly distributed in the monitored water area, and the position of the sink node is (75, 75, 75). In the MATLAB simulation environment, the performance of the DC-KH algorithm is verified. Table 1 shows the parameters used in the simulation experiment.

**Figure 2:**
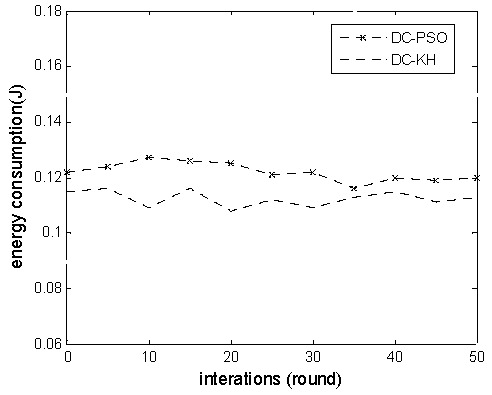
Energy consumption comparison chart of cluster head.

Figure 2 shows the DC-PSO and DC-KH algorithms with the number of rounds for increasing the cluster head node energy consumption changes. The graph particularly shows that, in each round of operation, the DC-KH algorithm cluster head consumption is always less than the DC-PSO algorithm. The reason is the fact that the DC-KH algorithm uses non-uniform clustering and the dynamic hierarchical mechanism based on the krill swarm optimization algorithm to select the master cluster heads and vice cluster heads. It avoids the repeated selection of nodes as the cluster head, and it improves the early energy utilization rate. Compared with the DC-PSO algorithm, the reasonable selection of the vice-cluster head decreases the partial burden of the master cluster head. In addition, a suitable multi hops routing path is selected by considering the communication overhead and energy consumption of the sub-cluster head. This process can help reduce the energy consumption of the cluster head effectively. In sum, the DC-KH algorithm cluster head energy consumption is lower than DC-PSO.

**Figure 3:**
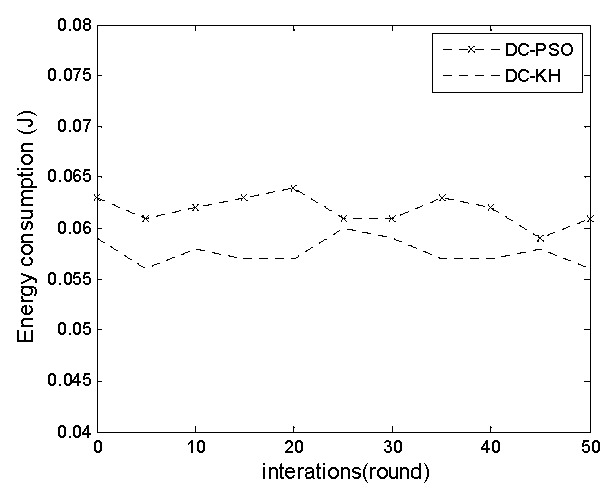
Energy consumption comparison chart of the main-cluster head.

**Figure 4:**
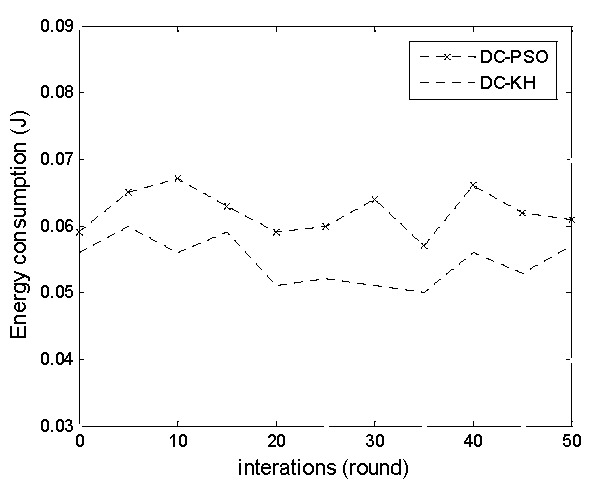
Energy consumption comparison chart of the vice cluster head.

Figure 3 and Figure 4, respectively illustrate the DC-PSO and DC-KH algorithms in the master cluster head and vice-cluster head energy consumption changes with the increase in the number of rounds. The graphs particularly show that regardless of the number of changes, the energy consumption of the master cluster heads and vice-cluster heads of the DC-KH algorithm is less than that of the DC-PSO algorithm. Krill swarm optimization method is adopted in the DC-KH algorithm to select the suitable master cluster heads and vice-cluster heads. The master cluster head collects the data. The vice cluster head reduces the energy burden of the master cluster head. Then, it transmits information to the sink node through a single hop or multi hops. The krill swarm optimization method using the Lagrange model can make the node orient to a high likelihood region. It is conducive to the choice of the master cluster heads and vice-cluster heads. Additionally, it reduces the energy consumption of master cluster heads and vice-cluster heads.

**Figure 5:**
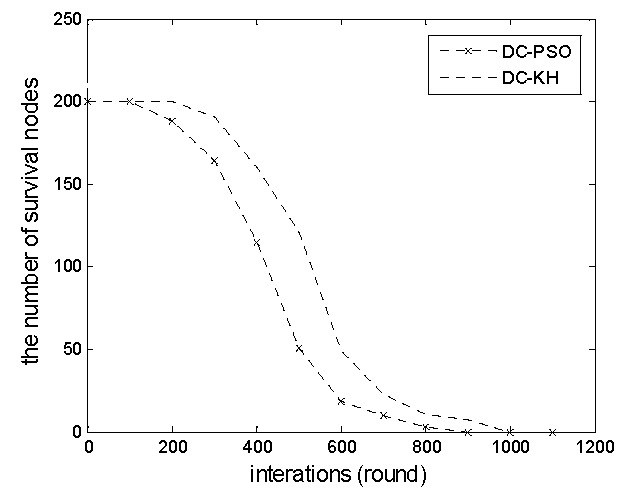
Comparison of the number of survival nodes.

Figure 5 shows the number of nodes for increasing the number of changes in the network in the DC-PSO and DC-KH algorithms. In this paper, the life cycle is defined as the time from the network operation to the node failure “death.” As shown in the figure, the life cycle of the DC-KH algorithm is longer than that of the DC-PSO algorithm. The reason is that the node in a different network running round number is selected as the optimal candidate cluster head repeatedly in DC-PSO. It leads to the rapid energy consumption of cluster head nodes, so nodes are susceptible to premature death. To compensate for this shortcoming, the DC-KH algorithm uses a dynamic hierarchical mechanism. Nodes are circularly selected as the cluster head. This procedure not only balances the energy consumption of the network nodes, but effectively prolongs the survival time of the network as well.

**Figure 6:**
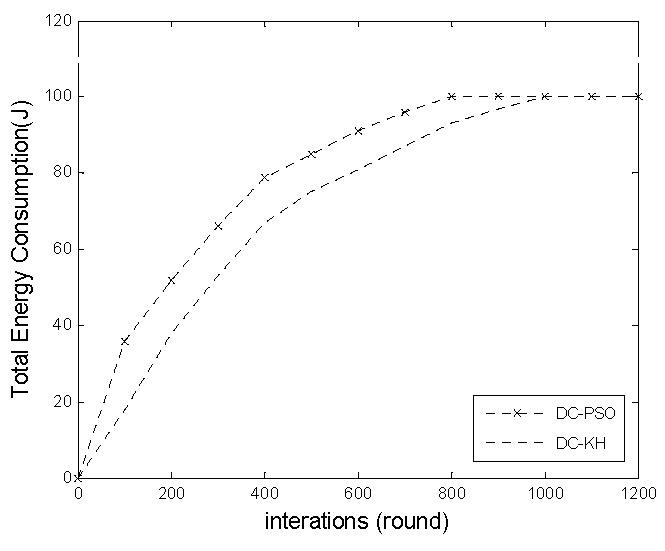
Network total energy consumption comparison chart.

Figure 6 demonstrates the DC-KH and DC-PSO algorithms in the network total energy consumption with the change of the number of rounds. The graph shows that the total energy consumption of the DC-PSO algorithm is always higher than that of the DC-KH algorithm. The proposed DC-KH is based on DC-PSO using non-uniform clustering and a dynamic hierarchical mechanism, which collaboratively addresses the network “hot spots”. In addition, the appropriate master cluster heads and vice-cluster heads are selected through the krill group optimization selection principle. The integration of the above three procedures makes the total energy consumption of the network DC-KH algorithm to be significantly less than that of the DC-PSO algorithm, and it prolongs the network running time of the algorithm.

**Figure 7:**
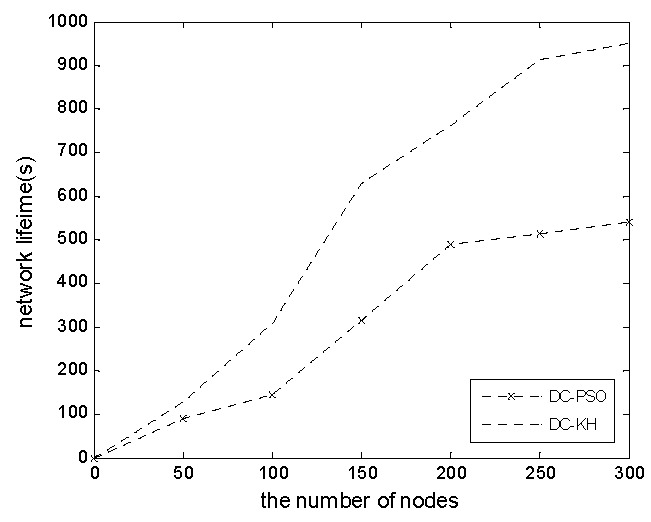
Network life cycle comparison chart.

Figure 7 shows the network survival cycle in the DC-KH and DC-PSO algorithms with the increase of the number of nodes. Network lifetime is an important basis for measuring the effectiveness of an algorithm [15]. In this paper, network lifetime is defined as the number of rounds that can satisfy the network coverage rate C_or_ (C_th_ ≤ C_or_ ≤ 100%). C_th_ is the coverage threshold. When the coverage rate is lower than the threshold value, the network can hardly complete the normal monitoring function and the end of the life cycle. The graph shows that the DC-KH algorithm is always higher than the DC-PSO algorithm in terms of network lifetime, because the dynamic hierarchical mechanism of DC-KH balances the cluster head energy consumption and prolongs the network lifetime.

## 5. Summary

A node’s premature death causes a burden on cluster heads in UWSNs and energy is unbalanced caused by network “hot spots”. To solve these problems, this study proposes a dynamic layered dual-cluster heads routing algorithm based on Krill Herd optimization in UWSNs. It uses non-uniform clustering, a dynamic hierarchical mechanism, and Krill Herd optimization method to choose the master cluster heads and vice-cluster heads. The developed algorithm can effectively solve the specified problems, it also prolongs the network life cycle. Considering the different sizes of clusters, future works should develop and implement different cluster head setting mechanisms.

## Figures and Tables

**Table 1:** Simulation parameters.

Parameter	Value
Initial energy (*J*)	0.5
Data packet	4000
Control packet	100
Iteration	5 TDMA
Moving speed δ (m/s)	1
Energy diffusion factor *k*	1.5
Communication radius R_t_	30
*α*	0.3
*β*	0.5
f (kHz)	10
τ	0.4
ε	0.6
$D^{max}$ (m/s)	0.005
∆t (s)	8
$I_{max}$	10
